# Dr. Paris, Dr. Beck, Dr. Smith, Dr. Male and Others, on Infanticide

**Published:** 1824-09-01

**Authors:** 


### MEDICAL JURISPRUDENCE.

### Infanticide.

1. *Medical Jurisprudence.* By Dr. Paris and Mr. Fonblanque. Three volumes, 8vo. 1824.
2. *The Principles of Forensic Medicine, systematically arranged and applied to British Practice.* By John Gordon Smith, M.D. Lecturer on Political Medicine. One volume, 8vo. Second Edition, greatly enlarged. 1824.
3. *Elements of Medical Jurisprudence.* By Theodore Romeyn Beck, M.D. Professor of the Institutes of Medicine, &c. in the State of New York. Two volumes, 8vo. Albany, 1823.
4. *Elements of Juridical or Forensic Medicine, for the Use of Medical Men, Coroners, and Barristers.* By George Edward Male, M.D. &c. &c. Second Edition, pp. 278. 1818.

Our readers will perceive, that since we commenced our chain of analytical articles on medical jurisprudence, the works on this subject, in the English language, have multiplied fast. Of

Dr. Paris's work, we have already given a pretty full account of the first and part of the second volume; and of the first edition of Dr. Smith's publication, we had occasion to make honorable mention in a former number of this Journal. The *second edition* is now before us, considerably enlarged and improved. It may, therefore, be considered as a standard work of its kind, intermediate, in size, between the works of Drs. Paris and Beck, and well calculated for a large proportion of the profession, in consequence of being portable, cheap, and unencumbered with purely legal discussions. The third on our list, the stranger from America, is really a very respectable performance, and nearly as much to our taste, as any thing we have yet seen on the subject of medical jurisprudence. The fourth work is well deserving of our notice, as being, till lately, the best work of the kind in the English language. It is said, indeed, to be too concise; but when we consider, that large and expensive books are seldom read by the great body of the profession, we are disposed to think, that Dr. Male's work has proved eminently *useful* as coming within the easy grasp of all. But as criticism forms an extremely small portion of our business, we shall not waste time, in attempting any comprehensive comparative view of the works before us, which could lead to no practical utility in respect to our readers, and would consequently be at variance with the "ruling passion" of our Journal. In this and our future numbers, we shall select particular subjects of medical jurisprudence for analytical articles, of greater or less extent, taking some *one* of the three works for our principal guide, and drawing information from the others as we see occasion.

In the present instance, we shall give the preference to our transatlantic author, not only from courtesy but from convenience; for we hope we are too free from narrow national prejudice, not to see merit because it happens to be on the North or South side of a channel, or on the East or West side of an ocean. Dr. Beck, indeed, has had good sense enough, not to take the petty effusion of one or two narrow minded individuals as specimens of a whole nation's feeling—and, therefore, he has treated the writings of his brethren on both sides of the Atlantic with candour, liberality, and good nature—a proper example for all writers in all countries.*

* The reason why we take Dr. Beck's for our guide is *not* because it is superior to the others, (in several instances it is inferior) but because its arrangement enables us to collate and avail ourselves of the information contained in Dr. Paris and Dr. Smith's publications.

### Infanticide—Fœticide—Prolicide.*

Dr. Beck (or rather his brother, to whom he is indebted for the chapter on infanticide) discusses his subject under five heads:—1st. The history of infanticide—2d. Fœticide, or criminal abortion—3d. Infanticide, or the murder of the child after birth—4th. The method of conducting the anatomical investigation of infanticide—5. The prevention of infanticide, with an examination of the effects of foundling hospitals. We shall endeavour to take a rapid view of each of these.

1. *History of Infanticide.* The love of progeny is so deeply implanted in human nature—indeed in all animated nature, that it seems incredible so forcible a law should ever be broken. Its breach proves that *artificial* feelings will sometimes conquer the strongest *natural* impulses or instincts. Thus, fear of the finger of scorn or ridicule, will induce a mother to imbrue her hands in the blood of her smiling infant!

In the early history of the Jews, infanticide does not appear to have been known—but subsequently, after intercourse with surrounding nations, the sacrifice of children took place. The Canaanites sacrificed their sons and daughters unto devils. Instances are not wanting among the Egyptians of great cruelty towards children, witness Pharaoh's bloody edict to the midwives. Towards their own offspring, however, they were, in general, very humane. Among the ancient Persians the inhumation of living children was common—and in most of the Grecian States infanticide was not merely permitted, but actually enforced by the government. Who knows not the Spartan law, that consigned all weakly or deformed children to the deep cavern at the foot of Mount Taygetus? The horrible edict of the Spartan Legislator was sanctioned by the most profound philosophers—the wise Aristotle—the mild and humane Plato! The Roman fathers were invested with an absolute authority over the lives and fortunes of their children; and history shews us how frequently they availed themselves of this prerogative. Fœticide was notoriously prevalent among the Romans—

---

* *Prolicide* is a term introduced by Dr. Smith, and we think with propriety, as signifying the murder of offspring generally; it is the converse of parricide, or the murder of our parents.

Dr. Paris has devoted about 46 pages, Dr. Smith 80, and Dr. Beck 104 pages to the subject under discussion.

Sed jacet aurato vix ulla puerpera lecto,
Tantum artes hujus, tantum medicamina possunt,—
Quo steriles facit, atque homines in ventre necandos
Conducit.—*Juvenal.*

Pliny defends the right of parents to destroy their children, as necessary to keep the increase of population within bounds: Malthus goes on the principle of *prevention*—" principiis obsta." Christianity first checked infanticide in Ancient Rome, as it still does in Modern Europe. But lawful infanticide was not confined to the Ancients. It still disgraces Eastern and Southern Asia. Nine thousand children are annually exposed in the streets of Pekin. We have seen them in the streets of Canton early in the morning—and floating down the Tigris in front of that city in wicker baskets. Among the Hindoos the horrible custom of infanticide is incorporated into their system of religion—or rather superstition. " The blood of their infants (as Dr. Beck forcibly expresses it) seems to have quenched completely the flame of humanity, while the lights of reason and truth are extinguished by the absurdities of superstition." They urge, in excuse, the difficulty of rearing female children, the expense of their education, and the small probability of their getting married. Two modes of infanticide (according to Buchanan) are practised. One consists in the introduction of a piece of opium into the mouth of the female infant—the other is strangling, by means of the umbilical cord round the neck of the child!

Till lately the Otaheiteans were not behind the Chinese and Hindoos in the practice of infanticide. Christianity has checked it there also. But it is unnecessary to extend this slight historical sketch any farther. It is somewhat to the honour of Europe, and the age in which we live, that the act of infanticide is a punishable crime by the laws of the civilized world—and, consequently, that it is only committed in solitary instances, in secret, and when the mind is torn with conflicting passions.

2. *Crime of Fœticide.* It is hardly necessary to say, that the law of the land is most absurd when it makes a difference in the punishment—and, consequently, in the degree of turpitude, between fœticide at three, and fœticide at five months utero-gestation—in other words, before and after quickening. Every one knows there is no just foundation for this distinction.

When a medical man is called in upon an investigation of this kind, the first thing is to ascertain whether or not there has been an abortion, whether natural or artificial. In the early months of pregnancy this point is with difficulty determined. The attachments of the fœtus to the uterus are slight —the constitutional changes unimportant—the hæmorrhage trifling. But in the middle and ulterior stages of pregnancy, the characteristics are less equivocal. There is considerable hæmorrhage—an offensive discharge from the vagina, which is dilated—the os uteri is open—the breasts are swollen—the abdomen flaccid and pendulous. The following appearances will be recognized on dissection, in case the mother dies.

"The uterus is found enlarged and thickened—its muscular fibres are more evident, and its blood vessels and lymphatics much augmented in size—a rough surface is found where the placenta has been attached—the cervix uteri is relaxed, and the vagina considerably dilated—the ligamenta rotunda are relaxed, and the ligamenta lata nearly effaced, as they furnish the uterus with its external covering. Upon examining the ovaria, if it be done a short time after the ovum has escaped from them, a *corpus luteum* is generally found, which vanishes soon after, but leaves a scar for life." 204.

The causes or agents of artificial abortion (for it would be needless to advert to natural abortion) are medicinal and instrumental. We believe, it is now pretty generally allowed, that there is no medicine which has the specific power of causing abortion. Drastic purgatives, emetics, diuretics, emmenagogues, or repeated venesection may, indeed, so derange the general health and disturb the functions of the system at large, as to induce abortion; but even this is very precarious—and endangers the mother's life as much as that of the infant. Savine and colocynth have had reputation in this way, but their effects are very uncertain, and at all events dangerous. Of all internal medicines the secale cornutum, or ergot of rye, is that most likely to produce the effect in question—but not, we imagine, without great danger to the mother.

Of the instrumental means, there is but one, which is at all safe; and that must be in the hands of the able surgeon. It is, of course, the rupturing of the membranes, as practised for premature delivery in cases of distortion.

"At Durham assizes, in 1781, Margaret Tinckler was indicted for the murder of Janet Parkinson, by inserting pieces of wood into her womb. The deceased took her bed on the second of July, and from that period thought she must die, making use of various expressions to that effect. She died on the 23d. During her illness, she declared that she was with child by a married man; and he, being fearful, should she be brought to bed, that the knowledge of the circumstance would reach his wife, advised her to go to the prisoner, who was a midwife, to take her advice how to get rid of the child—being at the time five or six months gone. The delivery took place on the 10th of July, three days previous to which, a person saw the deceased in the prisoner's bed-chamber, when the prisoner took her round the waist, and shook her in a violent manner five or six different times, and tossed her up and down. She was afterwards delivered at the prisoner's house. The child was born alive, but died instantly, and it was proved by surgeons to be perfect. There was no doubt but that the deceased had died by the acceleration of the birth of the child; and upon opening the womb of the mother, it appeared that there were two holes caused by wooden skewers, one of which was mortified, and the other inflamed. Additional symptoms of injury were also discovered."* 217.

It would appear that this delicate operation was not unknown to the ancients. Dr. Paris quotes a passage from Ovid which certainly seems to bear on this subject—

> " Vestra quid effoditis subjectis viscera telis,
> " Et nondum natis dira venena datis?

Tertullian reprobates the practice, and describes the instrument by which it was put in execution.—" Æneum spiculum quo jugulatio ipsa dirigitur, &c."

In concluding the subject of abortion Dr. Beck makes a remark (in the justice of which we are inclined to coincide) upon the *circumstantial evidence* which may be adduced to prove the guilt of the accused.

" With regard to her concealing her pregnancy, I cannot conceive with what justice any inference can be drawn prejudicial to her character. If her pregnancy be the result of illicit commerce, it is perfectly natural that she should make use of every effort to conceal her disgrace as long as possible. The mere fact of concealment, even if proved, ought to be considered as no evidence whatever of her guilt." 221.

We cannot conceive any thing more cruel than the old Scotch law which made the mere fact of concealing the pregnancy, whether the death of the child were proved or not, *a capital felony!* Although this law was repealed in 1803, there is still a punishment attached to the concealment.†

3. *Infanticide.* This is, of course, the destruction of the child after its existence has become independent of the mother.

* East Crown Law, vol. 1, p. 354. Smith, p. 306.

† We agree with Dr. Smith (2d. ed. p. 332) that the deed of infanticide is frequently the result of insanity, and that a verdict to this effect might be returned, in many cases of this kind, with at least as much truth, as in some cases of suicide. It is vain to urge that the insanity here is not real because temporary, while temporary insanity is readily admitted in cases of suicide.

There is reason to believe, as Dr. Paris has justly remarked, that previously to the time of Dr. William Hunter, many unfortunate women had fallen innocent victims of false theory and prejudice. That distinguished physiologist raised a host of objections against the validity of certain physiological tests of infanticide—objections which have probably operated in swaying the mind from credulity to the opposite extreme of scepticism, and thus brought unmerited distrust on the science of physiology. It is evidently, the wish of the three distinguished writers before us to rescue physiology, as far as possible, from this odium; and restore a certain degree of confidence, at least, to the tests which have been so much depreciated. The first medico-legal question is—*whether the child was born alive?* In a physiological point of view, we are to expect proofs of this in the circulating and respiratory systems :—we can expect little assistance from the nervous system. It is, of course, to be supposed that the child had come to its full period of utero-gestation, at least that it was rearable, or beyond the seventh month. After the above period, the fœtus will generally be found to weigh four pounds—though upon this, as upon most other subjects relating to infanticide, there is great diversity of opinion. Dr. Clarke found that the average weight, *at full term*, was seven pounds five ounces. In France the medium weight is said to be only six pounds and a quarter. In length, the fœtus is said to be about 20 or 21 inches, on an average.

" *Professor Chaussier* has presented us with a scale of relative admeasurements, from which he thinks we may deduce the age of a child. He asserts that at the full term of gestation, the middle of the body of the fœtus corresponds exactly with the umbilicus ; at the eighth month it is two or three centimeters higher ; that it approaches still nearer the sternum at the seventh month ; and at the sixth falls exactly at the abdominal extremity of that bone. If this statement is to be relied upon, we should be able to conclude, says *Dr. Smith*, that when the middle of the length of the body falls at the cartilago ensiformis, the fœtus must be under the seventh month, and consequently could not have continued to live after birth."* PARIS, p. 102.

* Some have supposed* that a perfect child at its full time should weigh eleven or twelve pounds, avoirdupois ; that a fœtus at eight months commonly weighs seven or eight pounds ; and at seven only about four pounds. Others† have estimated its weight at seven or eight, or at most ten pounds : others again as low as six or seven pounds, and its length from seventeen to twenty-one inches. Mr. Burns‡ says, that a fœtus in the sixth month, measures eight or nine inches, in the seventh about a foot, and in the eighth about fifteen inches. Dr. Hunter states¶ that of several thousand new-born

* Mahon. † Manuel d'Autopsie Cadaverique, p. 97. ‡ Principles of Midwifery, p. 121. ¶ Anatomical Description of the Gravid Uterus, p. 68.

*Proofs from the Circulation.* Before separate vitality, Becket found the blood (of animals) alike in the veins and arteries—and that similar to venous blood. He avers that he found the same appearances in the human fœtus in utero. In a chemical point of view, Fourcroy found the fœtal blood destitute of fibrinous matter, as well as of the phosphoric salts. He also found that it was incapable of becoming florid by exposure to the air.

In the fœtal circulation, it is evident that there is blood in the nutrient vessels only of the lungs. If therefore the other vessels be found distended with blood, it forms a reason for supposing the child had breathed. The *structure* of the lungs also will throw some light on the question. The lungs of the fœtus are small, dense, compact, of a deep red or chocolate colour, resembling liver. Their specific gravity (with some exceptions) exceeds that of water; and upon cutting into them no air will issue, nor blood either. These circumstances will be changed, if the child have breathed. The lungs become more voluminous, elastic, florid-red, containing blood and air—*swimming in water*. But this brings us to the *Opprobrium* Medicinæ Forensis, or the—

### HYDROSTATIC TEST.

This test, now so much doubted, once enjoyed the unreserved confidence of the public and of the profession at large. Medical evidence, on this point, was then very simple. In fact, a butcher was as good a witness as a physiologist. Whenever suspicion attached to the body of an infant, the lungs were taken out. If they sank the child was still-born—if they swam, then the child must have lived after birth—"quod signum ad infanticidia detegenda est evidentissimum." *Baglivi.* It was quite

---

and perfect children which were weighed at the British Hospital, the smallest was about four pounds, and the largest about eleven pounds two ounces; but by far the greater number were from five to eight pounds, avoirdupois. He adds, that he never knew an instance of a child born at the natural period weighing twelve pounds. Of sixty male and sixty female children, born at the full time, which were weighed by Dr. Clarke,* the lightest was four pounds, and the heaviest ten pounds; he found that the average weight of the males was seven pounds five ounces and seven drachms, avoirdupois; that of the females only six pounds eleven ounces and six drachms. The average weight of the fœtus at the full period, therefore, may be estimated at about seven pounds, avoirdupois. As it appears, then, that the weight of new-born children varies exceedingly, evidence founded on such uncertain principles ought to be very cautiously admitted in a court of justice.—*Male, p.* 152. *Second Ed.*

* Philosophical Transactions, 1786.

a witch's trial—and in both cases it is probable that justice did not always kick the beam. It is now well known that, under certain circumstances, the lungs of a fœtus born dead will swim in water, while those of a live-born child will sink in the same fluid. How many innocent females may have been sacrificed to a physiological error, God only knows!*

It is probable, however, that the profession and the bar have now run into an unwarrantable degree of scepticism on this point. Dr. Smith, one of our latest and best forensic writers, has strenuously advocated the general validity of the hydrostatic test—and that by examining the objections which have been made to it. These objections we will notice in as concise a manner as possible.

1. *It is objected that a child may have been born dead, and yet that the lungs may float in water, from the putrefactive process having commenced.*

Strange as it may appear, the putrefactive process, in lungs that have not respired, has been considered by some as rendering them *lighter*—by others, as rendering them *heavier* than water! Experientia fallax! Dr. Beck considers the experiments of Mayer as the most satisfactory on this litigated point.

"From a very extended series of experiments, continued during a number of years, and executed with the utmost care and precision, Mayer found, on putting into water the lungs of still-born children, in whom, of course, no sign of respiration or life had appeared, that they sunk to the bottom. After an interval of two or three days, the water in which they were left became turbid—the lungs changed in colour, and increased in volume—here and there an air bubble arose to the surface of the water, and at the same time a putrid odour became perceptible. All these appearances continued to increase daily, until the sixth, seventh, or, at the latest, the eighth day, when the lungs, both entire and cut into pieces, floated in the water in which they became putrid. On transferring the lungs to vessels containing clean water, they still continued to float, although on the slightest compression they instantly sunk.

"Lungs placed in water, and exposed to the rays of the sun, swam on the sixth day. If they were suffered to putrefy where there was a free current of air, they rarely floated before the tenth or eleventh day. After the lungs had once floated, they remained in that state, emitting

* Dr. Paris, who admits the great and serious objections to the hydrostatic test, must, we think, have made a mistake in the following passage. "It is now well ascertained, and as generally admitted, that the validity of the hydrostatic test, as usually applied, must afford very *unquestionable* indications." P. 110.

daily a more offensive odour, and acquiring an increased volume, until the twenty-first, or at the latest, the thirty-fifth day. After that period, they gradually sunk down, without a single exception, to the bottom of the vessel, nor did they afterwards betray any disposition to float, although kept for seven weeks, and in some instances a much greater length of time."* *Beck, p.* 230.

Dr. Beck made experiments of this kind himself, which were confirmatory of Mayer's. It may also be stated, that Haller made the same experiment, and came to the same conclusion.

"Bodies," says Dr. Gordon Smith, "have often been found in a very advanced state of putrefaction, where the lungs were not yet overtaken by it. Ballard was called to examine a child, the muscles of whose face were reduced to a pulp—to '*bouilli*,'—were in a state of putrefactive solution; and in which putrescence had advanced so far as to prevent discrimination of the sex; notwithstanding which the lungs immediately sunk.† These organs resist putrefaction longer than all the other parts of the body, excepting the bones. The fact has been accounted for by their compact structure previous to respiration, and their not having been excited to action, as well as to the impenetrable nature of the membrane in which they are enveloped, it being well adapted to oppose the passage of aëriform agents." *Smith, p.* 351. *2d. Edition.*

From the foregoing and other experiments, Dr. Beck comes to the conclusion, that, in the incipient stage of putrefaction, lungs that have never respired will float in water—whereas they will sink, if putrefaction has continued long enough to destroy their organization, and thus extricate all the air contained in them. This is familiarly exemplified in bodies that have been drowned. At first they sink—afterwards rise from putrefaction—and finally descend again, upon the extrication of the air. In what way then are we to discriminate between the floating of the lungs as caused by respiration—and that which is the result of decomposition?

"1. By the appearance of air bubbles on the surface of the lungs.

"On this subject, Dr. William Hunter lays down the following rule: 'If the air which is in the lungs be that of respiration, the air bubbles will hardly be visible to the naked eye; but if the air bubbles be large, or if they run in lines along the fissures between the component *lobuli* of the lungs, the air is certainly emphysematous, and not air which had been taken in by breathing.'‡ Jaeger had before this made a similar

* Mayer in Schlegel, vol. 1., p. 262, 3, 4.

† Translation of Metzger. See also Mahon, Med. Legale, II. p. 400.

‡ On the uncertainty of the signs of murder, in the case of bastard children. By William Hunter, M.D. F.R.S. Medical Observations and Inquiries of London, vol. 6, p. 284.

observation. In lungs floating from putrefaction, he describes the air as contained in the form of bubbles under the external membrane of those organs, where the air introduced by respiration never finds its way.* This rule appears to be founded in truth, and accordingly has been adopted by the best writers on forensic medicine.

"2. By the ease with which the air can be extricated from lungs which float in consequence of putrefaction. The evidence of this is to be found in the fact, that if lungs of this description, or any portions of them, be squeezed in the hand, they will immediately sink in water. On the contrary, no compression, however strong, can force out so completely the air from lungs that have respired, as to cause them to sink. This test is insisted upon by Marc, a very distinguished writer on this subject, as the most certain means of discriminating between the effects of putrefaction and respiration.†

"3. By cutting out a portion of the internal part of the lungs, and putting this in water, to ascertain whether it will float. If the lungs floated as the result of putrefaction, this internal portion will sink, inasmuch as the air generated by decomposition is confined to the surface of the lungs. If, on the contrary, the lungs have respired, the internal part will float more readily even than that towards the surface.

"4. By an examination of the other viscera of the body. Numerous observations have established the fact, that with the exception of the bones, the lungs resist putrefaction longer than any other part of the body. Faissole and Champeau, in experiments which they made upon drowned animals, observed that the lungs remained sound, after the whole of the body had become putrefied.‡ Mahon noticed the same fact in his dissections of dead bodies.§ Camper ascertained, by direct experiments, that the head became so far decomposed by putrefaction, that the slightest force was sufficient to detach the bones of it from each other, as well as those of the arms and legs, before the lungs began to participate in the putrefaction."|| *Beck, p.* 233.

2*dly. It is observed, that a still-born child's lungs may be inflated by the mother or others, after birth, and thus made to float in water.*

Dr. Smith admits the possibility of this event, and owns the difficulty of proving or disproving it physiologically. Dr. Beck, quotes a number of authors, as Heister, Hebenstreet, Roederer, who deny the possibility of inflating the lungs in this manner—and a still greater number of others, as Low, Bohn, Lutwig, Camper, Jaeger, Schmitt, and most of the French and English forensic writers, in favour of the artificial

* Schlegel, vol. 5, p. 111.
† Dictionaire des Sciences Medicales, vol. 10, art. Docimasie Pulmonaire.
‡ Mahon, vol. 2, p. 400. § Ibid. vol. 2, p. 400.
|| Dissertation on Infanticide, by W. Hutchinson, M.D. p. 47.

inflation, and consequent floating in water. But although the possibility or practicability of this event cannot now be doubted; yet we are inclined to agree with Drs. Beck and Smith, that it is not very likely to be effected by a female, under the distressing circumstances in which she would, at such a time, be placed. As for this inflation being performed by another, with the diabolical intention of criminating the mother, it is next to impossible, considering the cloud of doubt which now hangs over the hydrostatic test.

Dr. Beck, however, is confident that this artificial inflation may be distinguished from breathing, by one or more of the following tests.

1st. That of Buttner, founded on the difference between the fœtal and adult circulation. Thus, in the case of *inflation*, the vessels of the lungs would be empty—while, after natural breathing, they would be filled, in a certain degree at least, with blood.

2ndly. By Ploucquet's test, which is considered, by some, as an important one. Ploucquet found that the weight of the fœtal lungs (that is, before respiration) was to the weight of the whole body, as 1 to 70—whereas, the weight of the lungs, after respiration has commenced, is as 1 to 35. Thus, the tide of blood to the lungs, in consequence of the breathing process, or separate existence of the child, just doubles their weight.

As both Beck and Smith attach some importance to this test, and have treated largely upon it, we shall here dedicate a page to the subject.

In the first place, Ploucquet's trials were on too confined a scale to render the results certain. Lecieux, among 400 examinations, found such a discrepancy in the proportional weight of the lungs and body, as almost to discourage every expectation of establishing any ratio between them. According to the experiments of Chaussier, Schmitt, and Hartmann, the ratio was very different from that found by Ploucquet. The latter physician found (in the fœtus) as 1 to 59—and, after breathing, as 1 to 48. Here it is evident that the proportions are too nearly the same to permit our drawing any positive conclusions. Mahon and others have objected to this test, that such an excessive congestion of blood might take place in the lungs of a fœtus, *in utero*, as would render them equal in weight to the lungs of a fœtus which had breathed. This Ploucquet himself denies. But it is pretty certain, even from the experiments instituted by Dr. Beck himself, that putrefaction deranges the ratio between the weight of the lungs and body most materially. Thus, in two cases, where breathing had certainly taken place, Dr. B.

found the proportion as 1 to 35, and 1 to 37. In a case where the child was born dead, and where putrefaction had commenced, the proportion was as 1 to 46. In another case, where the child was born dead, and in an incipient state of putrefaction, but where the lungs were sound, the ratio was as 1 to 39. Thus, we see, that in this last case the proportion was almost exactly the same as in a child where breathing had been established. From the little that we have brought forward upon this subject, our readers will be inclined to acquiesce in the sentiment of Dr. Beck, that—"further observations are still called for, to settle the doubts occasioned by testimony so discordant and even contradictory."

The test of Daniel is somewhat similar to that of Ploucquet. He judges of the reality of respiration having taken place, from the increase of weight which a given quantity of water gains upon squeezing out the lungs into it. He thinks it may be known, also, from measuring the periphery of the thorax and lungs, and comparing their dimensions with those of an infant which has not respired. These tests, it is evident, are far inferior to that of Ploucquet.

The descent of the diaphragm, the diminution of the liver, the state of the bladder, &c. may assist in forming our opinion respecting the separate life of the child.

We now come to the various means by which a new-born infant may be destroyed.

These causes have been classed under the heads of *omission* and *commission*. The *former* includes those which prove fatal by the neglect of those precautions which every mother must feel are necessary at such a period—the *latter*, of course, embraces all acts of violence designed to destroy the life of the infant.

1. *Omission*. Death may be caused by omitting to remove the child from a state of supination, in which it may come into the world. Dr. Hunter relates the case of a child who died in consequence of its face lying in a pool made by the uterine discharges, and where no evil design could be imputed to the mother. Danger, or even death, may result from delay in administering nourishment to the newly born. Foderé says, a delay of 24 hours is dangerous. It is said that when death is occasioned by this cause, it may be known by the general emaciation of the body—by the fetid odour exhaled from it—by the eyes being open, and of a red colour; while dissection will shew the intestines completely empty—the gall bladder enlarged—the lungs withered, &c.

A great controversy once occurred respecting the fact of danger from neglect of tying the umbilical cord. It was urged that this danger was imaginary, since we see safe parturition take place every day among animals, without any ligature of the funis. But although a child is an animal, it does not follow that he is a calf. Professor Breudil found the umbilical vessels of the latter full of rugæ or folds, throughout their whole course—and in brutes, generally, he ascertained that the vessels of the cord were much smaller than in man, and that when the animal is born, they are, in a measure closed by a kind of cellular structure. Besides, in brutes, the cord is always torn, not cut, and it was wisely ordained that there should exist a peculiar construction of the vessels tending to interrupt the flow of blood through them, and favour their speedy contraction, when severed. This does not hold good, in the same degree at least, with man. It was designed that *he* should require assistance, in the early periods of his existence. If, therefore, it can be proved that the ligature of the funis has been wilfully neglected—unless through ignorance, as in a first pregnancy—it is fair to impute it to an intention of destroying the child. There have been cases, indeed, where separation of the cord has taken place without hæmorrhage; but these (as Dr. Smith properly observes) are only exceptions to general rules. Limbs have been shot and torn off, without any loss of blood.

2 *Commission.* The modes of infanticide under this head, are numerous. Thus, premature tying of the umbilical cord may be as fatal as total neglect of the ligature. Umbilical circulation, and pulmonary respiration, are vicarious functions; and if one be interrupted or destroyed before the other is in operation, death will be the consequence. The cord, therefore, should not be tied till breathing is established. Death, however, from this source, could scarcely result from other than ignorance. As for wounds and bruises, the same rules apply to children as to adults. It is necessary, however, to bear in mind that the heads of children are sometimes very much swollen from compression during a difficult parturition. This should not be confounded with that resulting from blows voluntarily inflicted. There have been, unfortunately, but too many instances of infanticide, by the introduction of needles and other sharp instruments into the head and other parts of newly born infants, as Guy Patin's History of the infamous Parisian Midwife exemplifies. In such cases, it is necessary to shave the head, when a slight ecchymosis will be found around the puncture.

*Suffocation.* Children are often suffocated by being placed under bedding, hay, chaff, mud, earth, &c. and, indeed, the mere application of the hand to the face of a helpless infant, will be sufficient to take away its life. Besides the usual marks of turgescence in the lungs, it will often be found that when an infant is smothered among any of the abovementioned substances, some particles may be detected in the mouth or nostrils. A careful examination of these parts should never be neglected.

*Drowning.* If a child have been immersed in water, the questions to be determined are—was it born alive?—was it put into the water before or after death?—The signs of drowning are precisely the same in the infant as in the adult, and need not here be discussed.

In respect to *hanging* and *strangulation*, the mark of the cord will be found round the neck—or ecchymoses, livor of the face, swelling and projection of the tongue, froth in the mouth, &c. will assist our judgments. On dissection, the vessels of the head will be found gorged with blood.* An objection lies against the signs above-mentioned—namely, that they may be the result of accidental strangulation, from the umbilical cord encircling the neck of the infant in utero.

These instances have undoubtedly occurred, but they are rare, and can only happen when the cord is of an extraordinary length.

In *these* cases the child cannot have respired, and therefore we have all the docimasial tests to assist us. In cases of wilful strangulation too, there will generally be found some marks of the fingers, or excoriations of the skin, both of which would be absent in natural strangulation by the funis.

The child may be suffocated by the turning back of the tongue upon the epiglottis—or life destroyed by twisting the head, so as to luxate and fracture the cervical vertebræ—by exposure to noxious airs—by poisons, &c.

But we must draw this short medico-legal sketch to a close, by an extract from Dr. Gordon Smith's "Practical Application," as laid in the second edition of his very excellent work.

"These great cavities being laid open, before we venture to handle any of their contents, we should take a general but an accurate view of their relative aspect; as regards the diaphragm, in particular, whether it be remarkably arched towards the thorax, or of the usual figure in the

---

* From the facts which will be found detailed in our review of Dr. Kellie's paper, it is doubtful whether the above phenomena be not the offspring of speculation in the closet, rather than actual anatomical investigations.—Rev.

bodies of those who have lived for some time. It will save inconvenience and promote accuracy, if the abdominal viscera be removed before those of the thorax be meddled with. First, however, let the position of the lungs be carefully remarked, how much of the cavities of the thorax they appear to occupy, likewise their colour, and general appearance in other respects. The liver should also be examined, and its sound or morbid state ascertained. The whole intestinal canal should be then removed, in the manner directed when treating of poisons.* Let the urinary bladder be examined as to its state in respect of distention or emptiness; and if any evacuation should have been caused by accidental interference, it is a circumstance that must not be left out of the account. The presence of fluids in the abdominal cavity must be looked for; and if there be any, their nature and origin should be verified—and any unusual or morbid appearance, as, for instance, of inflammation or lesion from violence, is of great importance.

"We now return to the thorax. It is impossible that the practitioner can forget the importance of the lungs, or overlook what is to be performed with them: but if he should inadvertently resort to one step of the process before another that should have preceded, he will mar the whole of his work—and in a court of justice will find himself in an awkward dilemma, being either compelled to make the humiliating admission of the truth, or reduced to shift unwarrantably the want of conclusions to the inapplicability of the subject—or, (which cannot be contemplated) draw such as are false. There is but one order in which the steps can be taken, and if, after having pursued the investigation fairly to the end, the result prove unsatisfactory, the professional witness will be at least able to speak boldly, and to maintain his own reputation.

"It will be necessary to ascertain whether there are adhesions between the lungs and the pleura costalis. If so, they must be noted, and separated by the finger with all possible delicacy. It is a principle to be strictly kept in view, that these organs are to undergo no more handling than is absolutely necessary. We now take out the lungs, separating them from the trachea as low as can be done with convenience; but as it is proper to preserve the heart in connexion with them in the first instance, ligatures must be placed upon the vena cava and aorta. Let the lungs be sponged clean, if covered with blood, and their consistence, soundness, and colour carefully verified. If any part seems morbid, or affected by putrefaction, let it be scrupulously noticed. They should be held inverted over a clean glass vessel, that if any fluid be ready to escape, it may be preserved. They are then to be weighed accurately, by drachms rather than ounces.

"A vessel about the size of a washing bason, and deeper, if it can be obtained, having been prepared, nearly full of clear fresh water, let the lungs be gently placed in it; and while they remain undisturbed, the following circumstances are to be carefully remarked: whether they sink or float—and if the former, whether they descend to the bottom—

* Article *Arsenic.*

rapidly or slowly—or, if they remain suspended *in* the water, at what depth. If they float, observe if the buoyancy is decided and general, or if one portion floats while another sinks. The result being recorded, place a ligature on the pulmonary vessels; separate the heart, by cutting between it and the ligature; reserve this organ for inspection; weigh the lungs alone, and place them a second time in the vessel of water. The appearances are again to be noticed in the same manner as before.

"If the corroborative proof of Daniel, mentioned above, can be performed, (and I see no reason for not adding it) the lungs are to be kept in the balance according to his instructions; but I would recommend the simplification of the experiment in this way—rejecting both the scales and the silver-wire basket for sinking the lungs. Let the practitioner place his hand in the water till it reaches any given height towards the arm, and mark the height to which the fluid is thereby raised upon the scale. Then let him sink the lungs under his hand, taking care to immerse it till the water reaches the same height as to his arm; and observe how much higher it stands then upon the scale. Solid bodies displace a quantity of water equal to their bulk.

"The next step in the process is to divide the right lung from the left, and to try them in the water separately. We must note any difference that appears in their degree of buoyancy; whether one sinks while the other floats; and if one floats more freely than the other it is of great consequence to ascertain whether it be the right or the left. The number and distribution of the lobes* should be remarked, whether there be three in the right lung and two in the left, or any variation from this the natural arrangement. The knife may now be applied, and when cutting through their substance we must be watchful for the crepitating sound that will issue from the cells, if they contain air, as well as to mark the appearance of hæmorrhage. Each lung, being cut in pieces, is to be tried thus in water, and any differences in respect of buoyancy are to be carefully noted. They are then to be pressed as forcibly as possible in the hand, or in a towel, and tried in water once more.

"The heart is next to be taken, and carefully inspected, beginning with the vessels. The ductus arteriosus should be laid open; and it is necessary to remark whether it contains blood, or is empty: the auricles and ventricles must be examined; and the circumstance of congestion there will excite suspicion of death by suffocation. The state of the foramen ovale lastly demands attention.

"It will be profitable to recapitulate here the import of the appearances we may suppose to have been discovered in the course of this investigation. If the diaphragm be very convex towards the thorax; and the lungs of a dark red colour, retracted from the anterior part of their cavities, not covering the pericardium, of a firm consistence, sink

---

* There is a every common *error*—for it goes beyond an inaccuracy—even among good writers, in regard to the lungs: they talk of the right and left *lobe*. It would not be much worse to say the right or left *lip*. We have two lungs as well as two eyes, but their lobes are no more designated right and left than the palpebræ.

in water under every variety of trial, emit no sound when cut into, and effuse no blood—when, along with these circumstances, blood is discovered in the ductus arteriosus, and the foramen ovale of the heart is open, the conclusion must be that respiration has never been performed. On the other hand, if we find that the lungs fill their cavities, are of a pink or light red colour, elastic to the touch, swim high in water, make a crepitating noise, and pour out florid blood on cutting into them, we have considerable proof that breathing has taken place: and if to these we should be able to add the corroborative result as to *absolute weight*, the mass of physiological evidence will be strong indeed. The mere fact of respiration not having been performed, is not, it seems, to be received as evidence that the child was not born alive. In this case all we can do is to declare that we can throw no further light on the matter from professional research, and leave it to law and justice to deal with the case in their own way. We should nevertheless continue the dissection, as we may, perhaps, ascertain more positively from other appearances, whether the child could have come into the world alive." 378.

In the above short article on infanticide, we do not pretend that we have done any thing like justice to the subject—indeed it would have been impossible in the narrow limits within which we were unavoidably placed. But we hope that, in the short space which we have occupied, we have crowded in as many of the more important facts and features of the discussion as could well be done under such circumstances—and that, by so doing from time to time, we shall draw the attention of our readers to, and keep it fixed upon, the important study of FORENSIC MEDICINE. Our reasons for so earnestly recommending this study, are not the same as those which forensic writers generally offer. The *forensic* application of these studies (very important no doubt in itself) we consider as chiefly useful in affording a new stimulus or impulse to the general study of anatomy, physiology, pathology, toxicology, &c.—for, in fine, FORENSIC MEDICINE embraces every department of the extended circle of medical science, and whoever is well grounded in his profession, will be a good forensic witness. We shall, on this account, select a forensic article for each Number of the Journal, in future, convinced that we cannot take a better mode of furthering the general progress of medical science, and the general interests of medical practitioners.*

* Just as this sheet was passing the press, we received the Philadelphia Journal of the Med. Sciences for May, 1824, in which Dr. Beck's work is reviewed. We were sorry to see the reviewer indulge in a sweeping (and we must say illiberal) reflection on the literary and moral character of the European Medical Journals and Journalists—a reflection which comes with a bad grace in a Journal that takes for its *detestation motto*, a somewhat

